# Electronic structure and thermoelectric properties of full Heusler compounds Ca_2_YZ (Y = Au, Hg; Z = As, Sb, Bi, Sn and Pb)†

**DOI:** 10.1039/d0ra04984k

**Published:** 2020-08-03

**Authors:** Yang Hu, Yurong Jin, Guangbiao Zhang, Yuli Yan

**Affiliations:** Institute for Computational Materials Science, School of Physics and Electronics, Henan University Kaifeng 475004 China yanyl@henu.edu.cn; International Joint Research Laboratory of New Energy Materials and Devices of Henan Province China; Chongqing Institute of Engineering Chongqing 402360 China

## Abstract

We investigate the transport properties of bulk Ca_2_YZ (Y = Au, Hg; Z = As, Sb, Bi, Sn and Pb) by a combination method of first-principles and Boltzmann transport theory. The focus of this article is the systematic study of the thermoelectric properties under the effect of a spin–orbit coupling. The highest dimensionless figure of merit (*ZT*) of Ca_2_AuAs at optimum carrier concentration are 1.23 at 700 K. Interestingly enough, for n-type Ca_2_HgPb, the maximum *ZT* are close to each other from 500 K to 900 K and these values are close to 1, which suggests that semimetallic material can also be used as an excellent candidate for thermoelectric materials. From another viewpoint, at room temperature, the maximum PF for Ca_2_YZ are greater than 3 mW m^−1^ K^−2^, which is very close to that of ∼3 mW m^−1^ K^−2^ for Bi_2_Te_3_ and ∼4 mW m^−1^ K^−2^ for Fe_2_VAl. However, the room temperature theoretical *κ*_l_ of Ca_2_YZ is only about 0.85–1.6 W m^−1^ K^−1^, which is comparing to 1.4 W m^−1^ K^−1^ for Bi_2_Te_3_ and remarkably lower than 28 W m^−1^ K^−1^ for Fe_2_VAl at same temperature. So Ca_2_YZ should be a new type of promising thermoelectric material at room temperature.

## Abstract 2

We investigate the transport properties of bulk Ca_2_YZ (Y = Au, Hg; Z = As, Sb, Bi, Sn and Pb) by a combination method of first-principles and Boltzmann transport theory.

## Graphical abstract

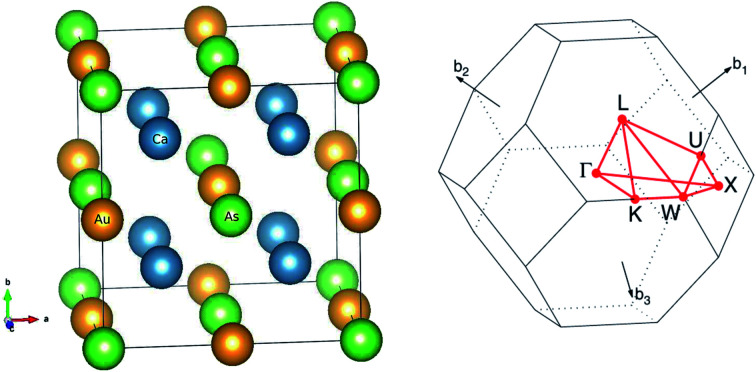

## I. Introduction

Thermoelectric (TE) materials, which can generate electricity from waste heat or be used as solid-state Peltier coolers, are considered for a variety of energy harvesting and thermal management applications.^1–3^ The efficiency of TE materials is described by the dimensionless figure of merit *ZT*, which is defined as *ZT* = *S*^2^*σT*/(*κ*_e_ + *κ*_l_), where *S* is the Seebeck coefficient, *σ* is the electrical conductivity (*S*^2^*σ* also known as the power factor, PF), *T* is the absolute temperature, and *κ*_e_ and *κ*_l_ are the electronic and lattice contributions to the thermal conductivity, respectively. Therefore, high thermoelectric performance requires both a high PF and a low thermal conductivity (*κ* = *κ*_e_ + *κ*_l_). There are two ways to improve the *ZT* of thermoelectric materials: one way is to enhance the PF, the other one is to suppress the thermal conductivity. Due to the strongly coupled electrical properties among *S*, *σ*, and *κ*_e_, tuning one of these parameters usually leads to a compensation in the others, resulting in the difficulty for enhancing *ZT*.

Heusler compounds have attracted renewed scientific interest because they have been expected to be new candidates for thermoelectric applications.^4–8^ Previous studies have shown that although Heusler compounds have high PF, high *κ*_l_ leads to a very low *ZT*. For example, the *S*^2^*σ* of Fe_2_VAl is as high as 4–6 mW m^−1^ K^−2^ (ref. 9–11) in the temperature range of 300 K to 400 K,^16^ the *S*^2^*σT* of TiIrAs, ZrIrSb, and ZrCoSb are more than 6 W m^−1^ K^−1^ at 800 K,^12^ but the higher *κ*_l_ seriously restricts the improvement of their *ZT* values.^9–13^ In recent years, more and more effects have been focused on lowering the *κ*_l_ to increase the *ZT* value by alloying, doping, and nono-structuring.^13–15^ However, these approaches may adversely affect the electronic transport properties. It is extremely expectant to find new thermoelectric materials with both the high PF and intrinsic low thermal conductivity. Excitedly, He *et al.* discovered a new family of FH compounds X_2_YZ (X = Ca, Sr, and Ba; Y = Au and Hg; Z = As, Sb, Bi, Sn and Pb) with ultralow *κ*_l_ by employing a high-throughput thermodynamic stability screening based on *ab initio* calculations. By comparing the PF of X_2_AuBi (X = Ba and Sr) with that of the well-studied thermoelectric compound Fe_2_VAl.^17–19^ They found that the maximum power factor of X_2_AuBi (X = Ba and Sr) is much larger than that of Fe_2_YZ.^17^ These findings suggest that FH X_2_YZ are very promising TE materials. Now we wonder why the author did not compare the PF of Ca_2_YZ (Y = Au and Hg; Z = As, Sb, Bi, Sn and Pb) with that of Fe_2_VAl.^17^ Could it be said that the PF of Ca_2_YZ is very low?

Considering that Ca is a earth-abundant, cheap and environmental friendly element, so in this article, we perform a comprehensive study on the electronic structure and the thermoelectric properties of Ca_2_YZ (Y = Au and Hg; Z = As, Sb, Bi, Sn and Pb) by a combination of first-principles calculations and the semiclassical Boltzmann theory. We compared the calculated PFS of Ca_2_YZ with that of Bi_2_Te_3_ (ref. 20) and Fe_2_VAl.^9–11^ The most significant thing is that at room temperature, the maximum value of PFS for Ca_2_YZ is PF ≧ 3 mW m^−1^ K^−2^, which is similar to that of ∼3 mW m^−1^ K^−2^ for Bi_2_Te_3_ (ref. 20) and ∼4 mW m^−1^ K^−2^ for Fe_2_VAl.^9–11^ And at room temperature, the theoretical *κ*_l_ of Ca_2_YZ is estimated to be about 0.85–1.6 W m^−1^ K^−1^.^17^ At the same temperature, the value is comparable to those of known thermoelectric materials *i.e.*, 1.4 W m^−1^ K^−1^ for Bi_2_Te_3_ (ref. 21) and 28 W m^−1^ K^−1^ for Fe_2_VAl^16^ at 300 K. So Ca_2_YZ should be a new promising material for thermoelectric applications.

## II. Computational detail

The crystal structures of X_2_YZ were fully relaxed using the Vienna *Ab initio* Simulation Package (VASP).^22^ The pseudopotential based on the projector-augmented-wave (PAW) method was used to describe the interaction between ionic cores and valence electrons, which is very accurate and efficient.^23–25^ The generalized-gradient approximation (GGA), as parameterized by Perdew, Burke, and Emzerhof,^26^ was used to describe the exchange–correlation interaction of electron. The cut-off energy of plane-wave basis sets is set at 500 eV, and the energy convergence criterion was chosen to be 10^−6^ eV. The Brillouin zone was sampled by the Monkhorst–Pack special *k*-point scheme with 14 × 14 × 14 grid meshes for Ca_2_YZ. All atoms are relaxed until the residual forces on each of them is smaller than 0.02 eV Å^−1^.

The electronic structures of Ca_2_YZ were calculated by the full potential-linearized augmented plane wave (FP-LAPW) methods^27^ based on the density functional theory (DFT),^28,29^ as implemented in the WIEN2k code.^30–32^ The muffin-tin radii (RMT) ware chosen 2.5 a.u. for all atoms. The calculations were performed with an energy cut-off such that *R*_MT_ × *K*_max_ = 7 and a *k* sampling with 14 × 14 × 14 Monkhorst–Pack mesh. Further increase in the cut-off value and the *k*-points number, the eigenvalues showed no significant change. The self-consistent cycles were stopped when the total energy difference between the cycles was less than 0.0001 eV. Since these compounds contains heavy atoms and so the scalar relativistic effect and spin–orbit coupling (SOC) effect are taken into account in the computations. Tran and Blaha-modified Becke–Johnson potential (TB-mBJ)^33,34^ with Perdew–Burke–Ernzerhof generalized-gradient approximation (PBEGGA)^35^ are used to obtain a more accurate value of the band gap.

The electrical transport coefficients are derived from the DFT electronic structure by using the Boltzmann theory within the constant scattering time approximation,^36–38^ as implemented in the BoltzTrap code.^39^ This approximation, which is commonly applied for metals and degenerately doped semiconductors,^40^ is based on the assumption that the scattering time determining the electrical conductivity does not vary strongly with energy on the scale of *kT*. It does not involve any assumption about the possibly strong doping and temperature dependence of *τ*. This method has been wildly used to calculate the transport coefficients of thermoelectric materials.^38,41,42^

## III. Results and discussion

### A. Crystal structure

**Fig. 1:**
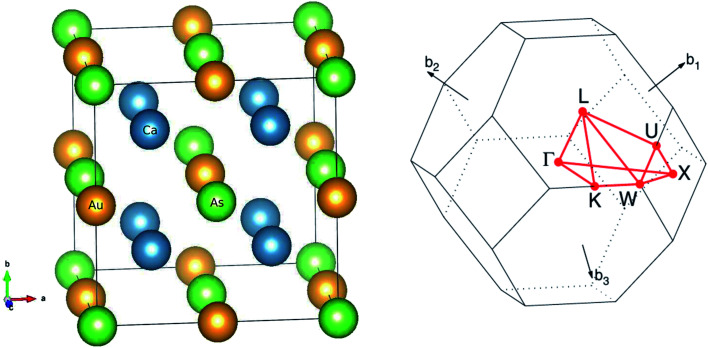
The optimized crystal structure of Ca_2_YZ (Y = Au, Hg; Z = As, Sb, Bi, Sn and Pb) in space group *Fm*3̄*m* and Brillouin zone.

The main focus of this study is a new family of intermetallic compounds with ten valence electrons Ca_2_YZ (Y = Au and Hg; Z = As, Sb, Bi, Sn and Pb), which was discovered through high-throughput *ab initio* screening.^17^ Most these compounds crystallize in the FH structure,^17^ which is a cubic lattice with space group *Fm*3̄*m* (no. 225). The Ca atom occupies the Wyckoff position 8*c* (1/4, 1/4, 1/4), Y and the Z atoms are located at 4*a* (0, 0, 0) and 4*b* (1/2, 1/2, 1/2), respectively. The FH structure can also be understood as the interpenetration of YZ face-center-cubic (FCC) and X_2_ simple cubic (SC) sublattices, as shown in Fig. 1. The conventional cell contains 16 atoms with eight equivalent calcium atoms. The Heusler structure exhibits high symmetry and large isotropy, indicating the same transport properties along the three principal axes of the crystal, so we calculated transport coefficient by averaging over three principal crystal axes.

### B. Electronic structure

**Fig. 2:**
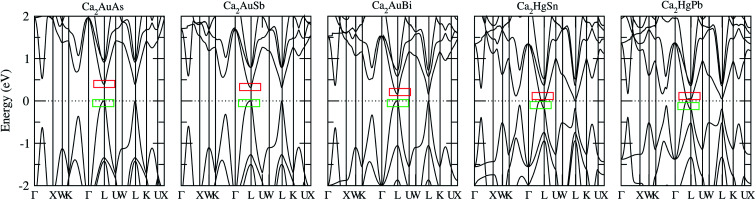
Calculated band structure of Ca_2_AuAs, Ca_2_AuSb, Ca_2_AuBi, Ca_2_HgSn and Ca_2_HgPb. The Fermi level is at 0 eV. Top of the valence band is set to zero on the energy scale; the red and green squares labeled in the band structure denote the carrier valleys for hole and electronic transport, respectively.

**Fig. 3:**
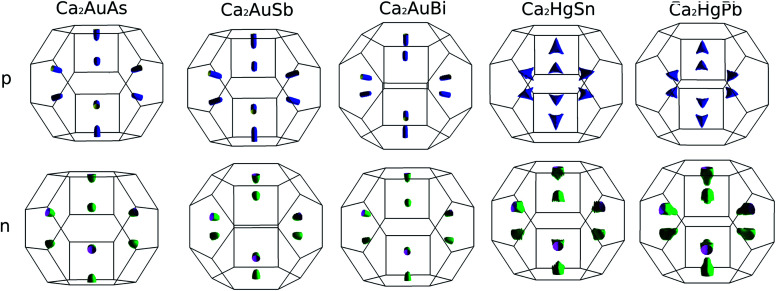
Fermi surface for a Fermi level 0.12 eV below the valence band maximum and above the conduction band minimum.

**Fig. 4:**
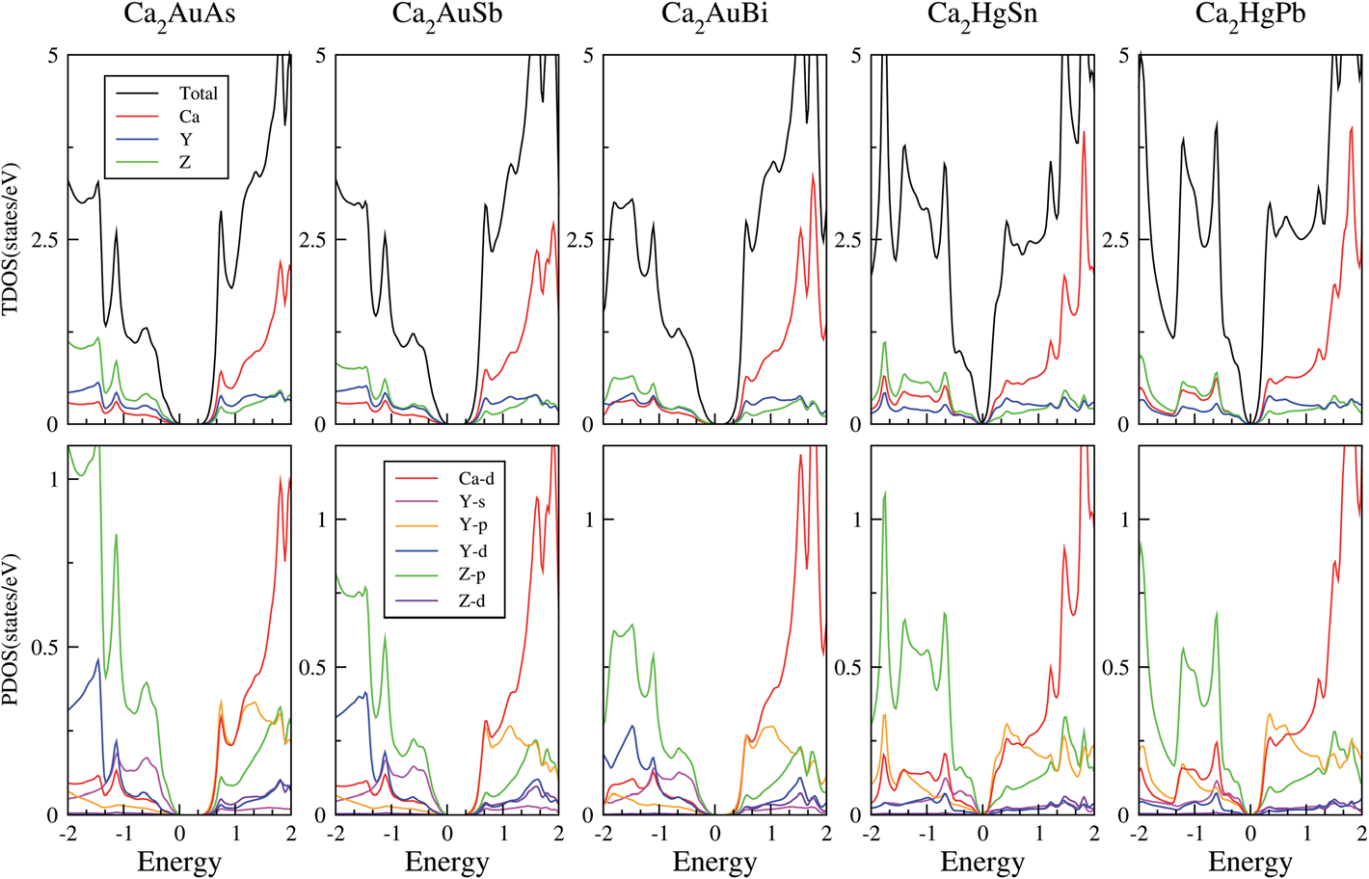
Calculated total, X-total DOS, Y-total and Z-total of Ca_2_YZ; the projected DOS of Ca_2_YZ. The Fermi level is at 0 eV.

To calculate electrical transport properties, an accurate electronic structure is required. The electronic structures of these FH compounds are calculated with FP-LAPW, TB-mBJ exchange potential, and spin–orbit coupling (SOC). Fig. 2 shows the calculated band structures of Ca_2_YZ (Y = Au and Hg; Z= Sn, Pb, As, Sb, and Bi). The band gaps of these compounds are 0.389, 0.313, 0.160, 0.00 and 0.00 eV for Ca_2_AuAs, Ca_2_AuSb, Ca_2_AuBi, Ca_2_HgSn and Ca_2_HgPb, respectively. Of particular note is that Ca_2_HgSn have metallic characteristic with very small cross band between the bottom of the conduction band and the top of the valence band. An overlap between hole and electron pockets gives rise to a semimetallic character for Ca_2_HgPb. All these band gaps are quite different from previous study,^17^ which is due to the different exchange-correction functionals and the contribution of SOC. We also note that both the valence band maximum (VBM) and conduction band minimum (CBM) of Ca_2_AuAs, Ca_2_AuSb, and Ca_2_AuBi are located at the *L* point, which has orbital degeneracy is 1. Considering the symmetry of the Brillouin zone, so the valley degenerate is 4. Interestingly enough, the VBM of Ca_2_HgSn and Ca_2_HgPb are also located at the *L* point and is flat along *L*–*Γ* direction, which possesses band degeneracy of *N*_v_ = 4, and CBM of Ca_2_HgSn and Ca_2_HgPb are located along *Γ*–*L* (*Λ*) line, which possesses band degeneracy of *N*_v_ = 8. A larger band degeneracy is favorable for large PF. In order to confirm the results, we calculated the isoenergy surface for a Fermi level 0.12 eV below the valence band maximum and above the conduction band minimum and shown in Fig. 3. As is clearly shown in Fig. 3, for Ca_2_AuZ (Z = As, Sb and Bi), the doped carrier arise from holes or electrons are located in small pockets centered at *L*, and associated with four fold degenerate valleys. While for n-type Ca_2_HgZ (Z = Sn and Pb), eight triangular cones along *Γ*–*L* connected by bottom angles exhibiting metallic behavior have a high degeneracy with 8. For p-type Ca_2_HgZ (Z = Sn and Pb), the center of the irregular isoenergetic surface exhibiting semiconductor behaviors is located at *L* point with larger pockets, which yields a four fold degenerate valence band edge. The number results are in good agreement with those obtained from band structures. We further show the total density of states (TDOS) and partial density of states (PDOS) of these compounds in Fig. 4. The TDOSs show that the conduction band of the these five compounds rises from the band edge much more rapidly than that of the valence band, which suggests that the absolute value of the Seebeck coefficient for electron doping should be significantly greater than that of hole doping. The top of valence band rises faster for Ca_2_HgZ (Z = Sn and Pb) than the other three compounds, which means the Seebeck coefficient for hole doping for Ca_2_HgZ (Z = Sn and Pb) is larger than that of the other three compounds. In fact, there are many factors affecting the Seebeck coefficient, such as the band gap, temperature, and carrier concentration. The analysis of PDOS further reveals the top of valence band is formed mainly by the Y s and Z p orbitals and the conduction band is composed of the Ca d and Y p orbitals. The two most electropositive atoms Ca donate their four 5 s electrons to the electronegative Y and Z atoms. The s states of the Z atom are extremely localized and far below the Fermi level, so it does not have contributions. These results can provide guidance for further investigating the doping effect on Ca_2_YZ with the appropriate atoms.

In order to have a deeper understanding of the electronic structure of Ca_2_YZ, we provide more detailed study in the following aspects. Firstly, we researched the differences in electronegativity between elements (as shown in Table 1). We can see that a large electronegativity difference between Ca and the Y/Z cite elements. It is expected, therefore, there is considerable charge transfer from the electropositive element Ca to the electronegativity Au(Hg) and As(Sb/Bi), indicating Ca_2_YZ are incline to be ionic compounds. However, the difference between Au and As(Sb/Bi) or Hg and Sn(Pb) is much smaller. So the type of chemical bond between Y and Z should be a polar covalent bond.

**Table 1:** The electronegativity of the elements (Pauling) (|*χ*|) and charge accumulation (*Q* > 0) and depletion (*Q* < 0) of the atoms in FH compounds Ca_2_YZ (Y = Hg and Au; Z = As, Sb, Bi, Sn, and Pb) based Bader charge analysis

Comp.	|*χ*_X_|	|*χ*_Y_|	|*χ*_Z_|	|*χ*_XY_|	|*χ*_XZ_|	|*χ*_YZ_|	|*χ*_X_2_YZ_|	*Q*_X_	*Q*_Y_	*Q*_Z_
Ca_2_AuAs	1.00	2.54	2.18	1.54	1.18	0.36	1.9067	1.404	−1.122	−1.687
Ca_2_AuSb	1.00	2.54	2.05	1.54	1.05	0.49	1.863	1.385	−1.123	−1.645
Ca_2_AuBi	1.00	2.54	2.02	1.54	1.02	0.52	1.8533	1.363	−1.185	−1.539
Ca_2_HgSn	1.00	2.00	1.96	1.00	0.96	0.04	1.6533	1.300	−0.917	−1.682
Ca_2_HgPb	1.00	2.00	1.87	1.00	0.87	0.13	1.6233	1.306	−0.972	−1.637

To further understand the mechanisms, we used Bader charge analysis^43^ to monitor changes in the charges on each atom of Ca_2_YZ compounds. As shown in Table 1, Ca apparently loses the charge, while both Y and Z gain electron, and the difference in charge obtained from X atom is not hug between the Y atom and Z atom: from 0.354 for Ca_2_AuBi to 0.765 for Ca_2_HgSn, which is consistent with the above analysis of electronegativity.

### C. Electrical transport properties

Based on the calculated electronic structures, the electrical transport properties of Ca_2_YZ (Y = Au and Hg; Z = As, Sb, Bi, Sn and Pb) are evaluated by using the semiclassical Boltzmann transport theory and the rigid-band model. The calculated results show that the absolute value and variation trend for the transport coefficient along the three main directions are almost identical, which is in accordance with the above discussion of crystal symmetry. Thus, in this paper, we only study the electrical transport properties along the *x* direction.

**Fig. 5:**
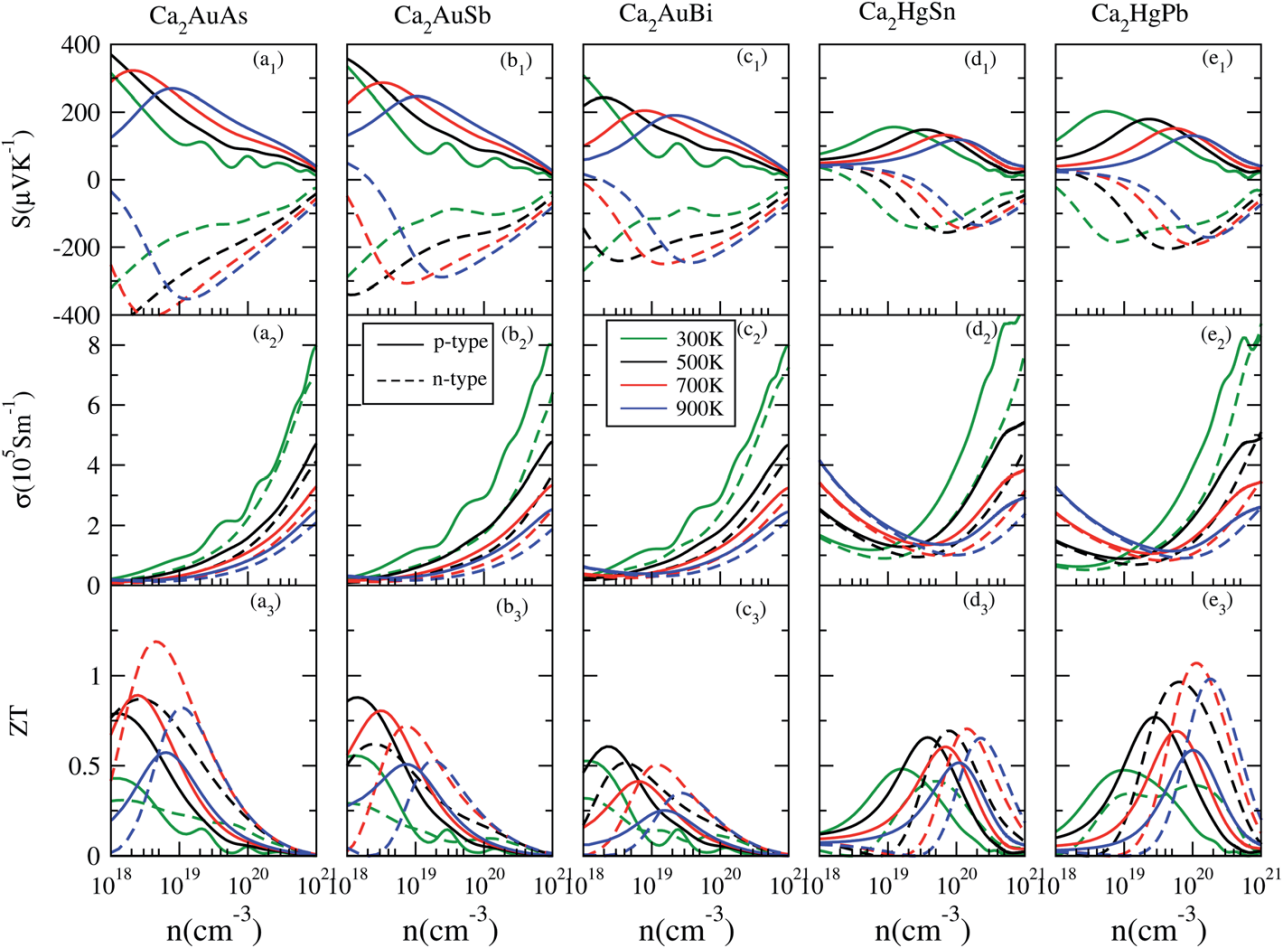
Transport coefficients of Ca_2_AuAs (a1–a3), Ca_2_AuSb (b1–b3), Ca_2_AuBi (c1–c3), Ca_2_HgSn (d1–d3) and Ca_2_HgPb (e1–e3) as a function of carrier concentration from 10^18^ to 10^21^ cm^−3^ at 300 K, 500 K, 700 K and 900 K.

Fig. 5 (a_1_–e_1_) show the calculated *S* as a function of carrier concentration at different temperature (300, 500, 700, and 900 K). We find that the *S* for Ca_2_AuZ (Z = As, Sb and Bi) is larger than that of Ca_2_HgZ (Z = Sn and Pb), which is mainly due to the metallicity of the latter, as we mentioned in the band structure analysis. It is worth mentioning that all the compounds have the maximal value of the absolute *S* greater than 200 μV K^−1^ except for that of Ca_2_HgSn. In further analysis, we found *S* decreases with the increasing of carrier concentration when at 300 K and 500 K, but increases at first and then decreases with the increasing of carrier concentration at 700 K and 900 K. It is worth noting that, the variation trend of *S* with temperature and carrier concentration for Ca_2_HgZ (Z = Sn and Pb) in the whole temperature range under study is quite similar with that of Ca_2_AuZ (Z = As, Sb and Bi) at 700 K and 900 K, which will be explained in the following. For a semiconductor with carriers including hole and electron, known as bipolar effect, the effective Seebeck coefficient (*S*_E_) is defined as:^44^1
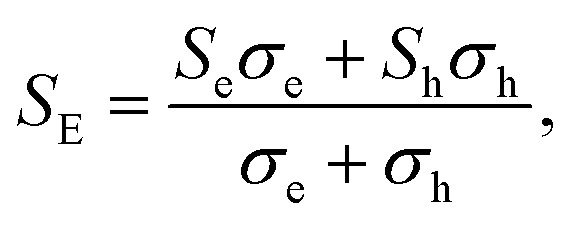
where *σ*_h_ (*σ*_e_) is the electrical conductivity of hole (electron), and *S*_h_ (*S*_e_) is the Seebeck coefficient of hole (electron). At lower carrier concentration and higher temperatures, bipolar effect caused by thermal activation has a relatively large influence on the *S*. The bipolar effect is becoming more and more serious with the decreasing of band gap, which leads to the decreases gradually of *S*, as shown in Fig. 5 (a_1_) to (e_1_).

Interestingly, the absolute value of the *S* for p-type and n-type Ca_2_HgPb are larger than that of Ca_2_HgSn, which is mainly due to the larger degeneracy in the second and third conduction bands at the *L*-point for Ca_2_HgPb than for Ca_2_HgSn. Hence, it is very important to optimize carrier concentration, engineer band gap, and align band edge to achieve the largest *S*.

Here, we discuss *S* from a different view, taking Ca_2_AuAs as an example. As Fig. 5 (a_1_) shows that at the same temperature, the maximum of |*S*| (absolute value of Seebeck coefficient) for n-type doping is greater than that of p-type, which can be explained as follows: the *S* of metals or degenerate semiconductors (parabolic band, energy-independent scattering approximation) is defined as:^45^2
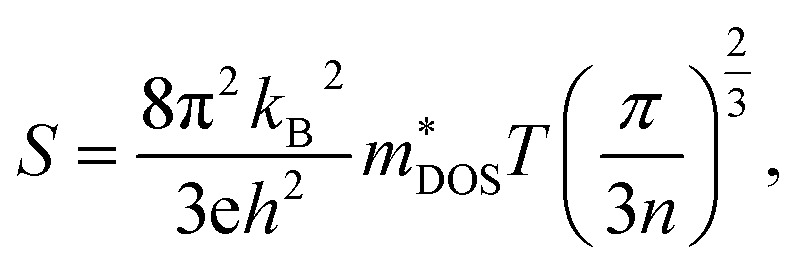
where *n* is the carrier concentration, *k*_B_ is the Boltzmann constant, *h* is the Planck constant, 
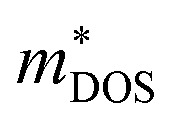
 is the density-of-states effective mass, which is defined as *N*_v_^2/3^ (*m*_b(*x*)_*m*_b(*y*)_*m*_b(*z*)_)^1/3^. For an isotropic material with a same band masses of *m*_b_ (*m*_b(*x*)_ = *m*_b(*y*)_ = *m*_b(*z*)_ = *m*_b_) along three principle directions, 
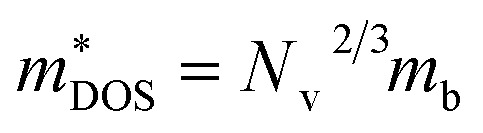
, where *N*_v_ is the band degeneracy of an energy band near the Fermi level. The above equation implies that *S* increases with the increasing of the slope of 
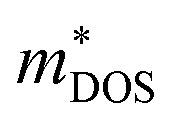
 under certain conditions of temperature and carrier concentration. In general, 
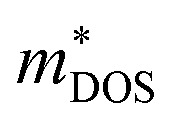
 is positively correlated to the steepness of the Fermi–Dirac distribution near the Fermi energy. And Fig. 4 just tells us that the steepness of the total DOS of the valence band near the Fermi energy level is larger than that of the conduction band, which could be another reason that the maximum |*S*| of electron doping is greater than that of hole doping.

In this section, we discuss the carrier concentration and temperature dependence of the electrical conductivity (*σ*). It is impossible to obtain *σ* solely using the electronic structure information since the relaxation time *τ* is unknown. To solve this question, we use the method of Ong and coworkers^46^ to eliminate the effects of *τ*. By comparing the experimental *σ* with our calculated *σ*/*τ* values at the same temperature and carrier concentration, we can obtain *τ*. However, there is little experimental research on Ca_2_YZ (Y = Au, Hg; Z = As, Sb, Bi, Sn and Pb), we are having to replace them with Fe_2_VAl.^47^ In ref. 47, the electrical resistivity to the experimental value of 0.65 mΩ cm for Fe_2_VAl_1*x*_M_*x*_ (M = Si, Ge) systems at doping *x* = 0.03 and 300 K, which combined with the calculated *σ*/*τ* yields *τ* = 3.62 × 10^−15^ s for Fe_2_V_1−*x*_Al_*x*_ at 300 K. Near the temperature, the experimental data for this sample and others follow an approximate electron–phonon temperature dependence 
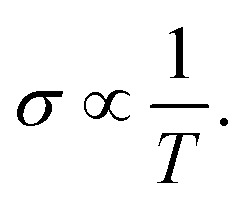
 On the other hand, we take into account the impact of doping on electric–phonon form. This yields *τ* = 8.73 × 10^−6^*T*^−1^*n*^−1/3^. We then estimate *σ* as 
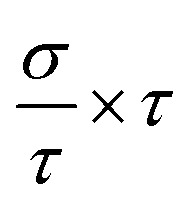
. Fig. 5 (a_2_–e_2_) show the relationship between the *σ* and the carrier concentration. At higher carrier concentration, regardless of n-type or p-type doping, electrical conductivities increase with increasing carrier concentration, which is in agreement with electrical conductivity proportional to the carrier concentration. These figures also shows that at the same doping concentration, the conductivity decreases with the increasing of temperature, which is due to the carrier mobility decreasing with the increase of temperature. However, at low carrier concentration, the electrical conductivities of Ca_2_HgZ (Z = Sn and Pb) increase with the increasing of temperature and the decreasing of carrier concentration, which may be due to the influence of minority carrier. It is worth noting that for the same carrier concentration and temperature, the electrical conductivities of Ca_2_HgZ (Z = Sn and Pb) are generally larger than that of Ca_2_AuZ (Z = As, Sb and Bi). This is a result of the semimetallic properties of Ca_2_HgZ (Z = Sn and Pb). Therefore, it is possible for semimetal materials to be good thermoelectric materials. It should be noted that we have calculated the electron transport properties by the method used in this paper, and the results show that good agreement is obtained between calculated and experimental values, which proves the reliability of our calculation.

We also calculated the PF of these materials. Results show that the maximum PFs at an optimal carrier concentration are more than 3 mW m^−1^ K^−2^, which is very close to that of ∼3 mW m^−1^ K^−2^ for Bi_2_Te_3_ (ref. 20) and ∼4 mW m^−1^ K^−2^ for Fe_2_VAl.^9–11^ On the other hand, the theoretical *κ*_l_ of Ca_2_YZ (Y = Au and Hg; Z = As, Sb, Bi, Sn and Pb) is estimated to be about 0.85–1.6 W m^−1^ K^−1^ (ref. 17) at 300 K. The value is comparable to those of known thermoelectric materials *i.e.*, 1.4 W m^−1^ K^−1^ for Bi_2_Te_3_ (ref. 21) and 28 W m^−1^ K^−1^ for Fe_2_VAl^16^ at the same temperature. So Ca_2_YZ should be a very promising material for thermoelectric applications.

### D. Thermal conductivity

A valid theoretical approach to compute the thermal conductivity in thermoelectrics is of tremendous importance in material optimization for efficient thermoelectric refrigeration and power generation. As mentioned above, thermal conductivity in materials comes from two sources: (1) phonons travelling by the vibrating lattice (*κ*_l_); (2) electrons and holes transporting heat (*κ*_e_):

3 *κ* = *κ*_l_ + *κ*_e_.

We adopt *κ*_l_ of Ca_2_YZ (Y = Au and Hg; Z = As, Sb, Bi, Sn and Pb) from ref. 17. The electronic thermal conductivity *κ*_e_ is calculated through the Wiedemann–Franz law:^48^

4 *κ*_e_ = *LσT* = *neμLT*

where *L* is the Lorenz constant.^30^ The value of the Lorentz constant is approximately ∼2.45 × 10^−8^ V^−2^ K^−2^ for metals or a degenerate semiconductor, and 1.5 × 10^−8^ V^−2^ K^−2^ for non-degenerate semiconductor.^49^ We take the value of the Lorentz constant as 1.5 × 10^−8^ V^−2^ K^−2^ for Ca_2_AuZ (Z = As, Sb and Bi)^50^ because of their semiconductor properties, and 2.45 × 10^−8^ V^−2^ K^−2^ for Ca_2_HgZ (Z = Sn and Pb) because of their metal properties.

### E. Optimized *ZT* value

With all of the transport coefficients available, we calculated the *ZT* values of Ca_2_YZ for different carrier-concentration at 300, 500, 700, and 900 K and shown in Fig. 5 (a_3_–e_3_). From these figures, we can see that the optimal *ZT* values of n-type Ca_2_YZ are larger than that of p-type except 300 K, although the electrical conductivities of p-type Ca_2_YZ are larger than that of n-type. The optimal carrier concentration for Ca_2_HgZ (Z = Sn and Pb) are larger than that of Ca_2_AuZ (Z = As, Sb and Bi). We also found that the magnitude of the optimal *ZT* values reduces firstly and increases afterward with the decrease of the energy gap. Very interestingly, the optimal *ZT* value for n-type Ca_2_HgPb is much larger than that of Ca_2_HgSn, which is mainly due to the absolute value of the Seebeck coefficient for n-type Ca_2_HgPb are larger than that of Ca_2_HgSn. It is noteworthy that the optimal *ZT* value for n-type Ca_2_HgPb is comparable with those of Ca_2_AuAs. And the values of maximum ZTs are close to each other at different temperatures and carrier concentrations, which provides ideal conditions for the application of Ca_2_HgPb in the thermoelectric material field. It's remarkable that the maximum *ZT* value of Ca_2_AuAs and Ca_2_HgPb at optimum carrier concentration are 1.23 and 1.1 respectively at 700 K.

## IV. Conclusion

In summary, we studied a new class of thermo-dynamically stable FH compound with excellent thermoelectric properties using the first principles calculations and semi-classical Boltzmann theory. Our calculation results show that the band gap decreases with the decreasing of the average electronegativity. The analysis of the band structure shows that Ca_2_AuZ (Z = As, Sb and Bi) exhibit semiconductor properties, however, Ca_2_HgSn and Ca_2_HgPb exhibit metallic and semimetallic characteristic, respectively. The thermally activated behavior has been clearly observed for Ca_2_HgSn and Ca_2_HgPb at low carrier concentration, pointing out the existence of a pseudogap at around the Fermi level, which has been confirmed by the analysis of the TDOS. It is worth noting that for the same carrier concentration and temperature, the electrical conductivities of Ca_2_HgZ (Z = Sn and Pb) are generally larger than that of Ca_2_AuZ (Z = As, Sb and Bi), which then lead to the close proximity of the thermoelectric conversion efficiency for n-type Ca_2_HgPb and Ca_2_AuAs. What's interesting is, for Ca_2_HgPb, the maximum *ZT* and the corresponding optimal n-type doping concentration are close to each other from 500 K to 900 K, which suggests that semimetallic material can also be used as an excellent candidate for thermoelectric materials. It is noteworthy that the maximum *ZT* value of Ca_2_AuAs and Ca_2_HgPb at optimum carrier concentration are 1.23 and 1.1 respectively at 700 K. On the other hand, at room temperature, the maximum value of PFS for Ca_2_YZ are greater than 3 mW m^−1^ K^−2^, which is equivalent to ∼3 mW m^−1^ K^−2^ for Bi_2_Te_3_ and ∼4 mW m^−1^ K^−2^ for Fe_2_VAl. And at room temperature, the theoretical *κ*_l_ of Ca_2_YZ is only about 0.85–1.6 W m^−1^ K^−1^, which is comparable to those of known thermoelectric materials *i.e.*, 1.4 W m^−1^ K^−1^ for Bi_2_Te_3_ and 28 W m^−1^ K^−1^ for Fe_2_VAl at 300 K. As a conclusion, it is reasonable to believe that Ca_2_YZ (Y = Au and Hg; Z = As, Sb, Bi, Sn and Pb) should be a very promising thermoelectric material.

## Author contributions

Yuli Yan carried out the calculations and is responsible for organizing the manuscript. Yang Hu and Yurong Jin contributed equally to this work. All authors participated in the discussion.

## Conflicts of interest

There are no conflicts of interest to declear.

## Supplementary Material

RA-010-D0RA04984K-s001
