# Supplementary material for: Electronic structure and thermoelectric properties of full Heusler compounds Ca2YZ (Y = Au, Hg; Z = As, Sb, Bi, Sn and Pb)
Source: RSC Adv. 2020 Aug 3;10(48):28501–8. doi: 10.1039/d0ra04984k (PMC9055840; doi:10.1039/d0ra04984k)
Supplement: RA-010-D0RA04984K-s001 [file RA-010-D0RA04984K-s001.pdf]

Electronic Supplementary Material (ESI) for RSC Advances.
This journal is © The Royal Society of Chemistry 2020

Supplementary Materials for

Electronic Structure and Thermoelectric Properties of Full Heusler Compounds Ca_2YZ (Y = Au, Hg; Z = As, Sb, Bi, Sn and Pb)

Yang Hu[1], Yurong Jin[1,3], Guangbiao Zhang[1,2], Yuli Yan[1,2*]

[1]*Institute for Computational Materials Science,*
School of Physics and Electronics, Henan University,
Kaifeng 475004, China;red[2] International Joint Research Laboratory
of New Energy Materials and Devices of Henan Province,
China; [3]Chongqing Institute of Engineering, Chongqing 402360, China

(Dated: July 20, 2020)

Introduction

To determine the phase stability, we calculated the phonon disperison using phonopy [1] and forces from vasp [2] in the frozen phonon approximation with 2×2×2 supercell. Phonon dispersion of Ca_2YZ (Y = Au, Hg; Z = As, Sb, Bi, Sn and Pb) along the high-symmetry direction is shown in Fig.S1. From this figure we can see that the calculated phonon spectra have no imaginary frequency, which indicates that Ca_2YZ (Y = Au, Hg; Z = As, Sb, Bi, Sn and Pb) are stable thermodynamically.

[1] Togo, A. Tanaka, I. First principles phonon calculations in materials science. *Scr. Mater.* **108**, 15 (2015).

[2] D. M. Ceperley and B. Alder, Ground state of the electron gas by a stochastic method, *Phys.Rev.Lett.* **45**, 566 (1980).

Table.S I: Total free energy of a f.u. Ca_2YZ (Y = Au, Hg; Z = As, Sb, Bi, Sn and Pb).

Comp.	Eg(eV)/f.u. (L21)	Eg(eV)/f.u. (XA)
Ca_2AuAs	-15.6702	-13.3465
Ca_2AuSb	-14.9002	-7.56019
Ca_2AuBi	-14.2891	-12.456
Ca_2HgSn	-10.5097	-8.9281
Ca_2HgPb	-9.9781	-8.5392

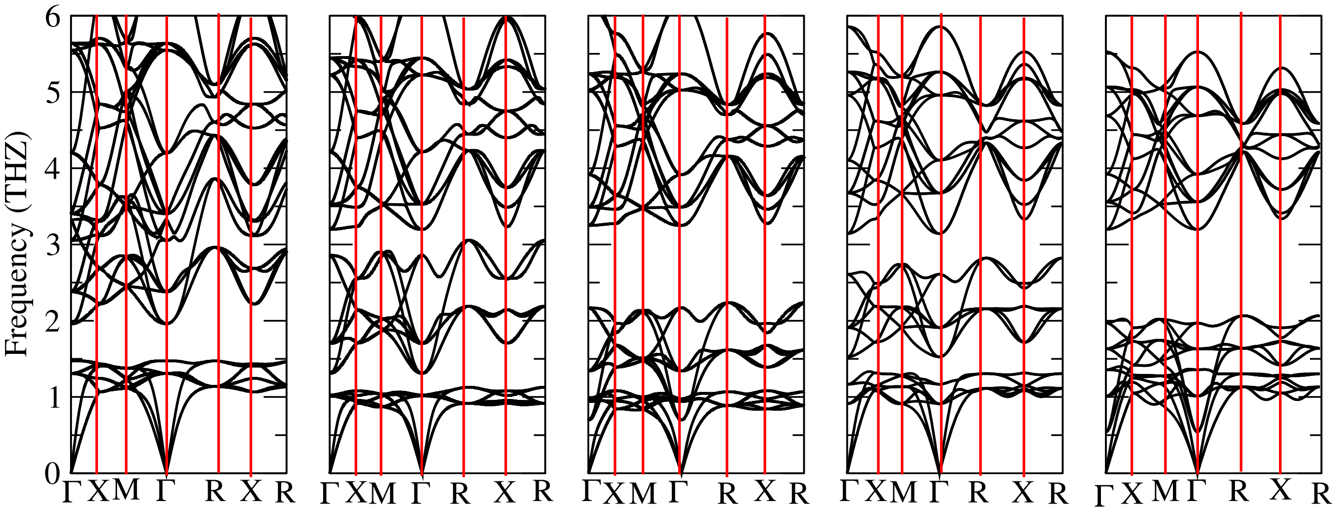

Fig.S 1: Phonon dispersion of Ca_2YZ (Y = Au, Hg; Z = As, Sb, Bi, Sn and Pb).
